# *Bambusimukaria*, a new bamboo-feeding leafhopper genus from China, with description of one new species (Hemiptera, Cicadellidae, Deltocephalinae, Mukariini)

**DOI:** 10.3897/zookeys.563.6030

**Published:** 2016-02-15

**Authors:** Lin Yang, Xiang-Sheng Chen, Zi-Zhong Li

**Affiliations:** 1 Institute of Entomology, Guizhou University, Guiyang, Guizhou Province 550025, P. R. China; 2 The Provincial Special Key Laboratory for Development and Utilization of Insect Resources, Guizhou University, Guiyang, 550025 P. R. China; 3 College of Animal Sciences, Guizhou University, Guiyang, Guizhou Province 550025, P. R. China

**Keywords:** Cicadomorpha, Oriental region, species diversity, taxonomy

## Abstract

A new genus and species, *Bambusimukaria quinquepunctata* **gen. & sp. n.**, feeding on bamboo in Guizhou and Fujian, China, are described and illustrated. The characters of crown, frontoclypeus, forewing venations and male genitalia place the new genus in the tribe Mukariini.

## Introduction

The bamboo feeding leafhoppers from China were reviewed by Chen et al. (2012). Four of the new species described in this work, i.e., *Abrus xishuiensis* Yang & Chen, *Bambusimukaria quinquepunctatus* Yang, Chen & Li, *Bundera bambusana* Yang & Chen and *Paraonukia wangmoensis* Yang & Chen, were stated as species in press. Although, for all intents and purposes, these species were well described in this work, they do not fit the criteria of the Code (Art. 16.1) in one respect: it was not the authors’ intention to formally describe them as new in that publication. Subsequently, the first of these species was named by Yang and Chen (2013) and the last two by Yang et al. (2013). It is the purpose of this paper to formally describe the fourth species *Bambusimukaria quinquepunctatus* and to also assign it to a new genus.

The tribe Mukariini was erected by Distant (1908), placed in the subfamily Nirvaninae (Evans 1947; Li and Chen 1999), and then raised to Mukariinae (Linnavuori 1979; Oman et al. 1990; Hayashi 1996). Recently, it was transferred to the subfamily Deltocephalinae based on molecular and morphological data (Zahniser and Dietrich 2010, 2013). The tribe contains the following genera: *Agrica* Strand, 1942 (three species); *Benglebra* Mahmood & Ahmad, 1969 (two species, reviewed by Khatri and Webb 2011); *Buloria* Distant, 1908 (one species); *Flatfronta* Chen & Li, 1997 (two species); *Mohunia* Distant, 1908 (six species, reviewed by Chen et al. 2007); *Mukaria* Distant, 1908 (13 species, reviewed by Yang and Chen 2011); *Neobassareus* Koçak, 1981 (nine species); *Neomohunia* Chen & Li, 2007 (one species); *Paramohunia* Chen & Li, 2007 (one species); *Pseudobalbillus* Jacobi, 1912 (18 species); *Pseudomohunia* Li, Chen & Zhang, 2007 (one speices); *Scaphotettix* Matsumura, 1914 (four species, reviewed by Dai et al. 2009); *Tiaobeinia* Chen & Li, 2008 (three species).

The following characters place the new genus in Mukariini: crown strongly sloping, frontoclypeus mostly flat, forewing venation obscure except near apex, with four apical cells and appendix well developed and aedeagus with paired shafts and two gonopores.

## Materials and methods

The study on bamboo leafhoppers in China was carried out from 2001 to 2011 for a minimum of ten weeks each year (June to October). All specimens were collected by sweep net in southern provinces of China and were counted and identified in the laboratory using a binocular microscope. A total of 8,000 leafhopper specimens from bamboo were examined and a total of 58 different genera and at least 123 species were identified, belonging to eight subfamilies (Chen et al. 2012).

In the present paper, terminology follows Li et al. (2011) except leg chaetotaxy, which follows Rakitov (1997). Dry specimens were used for the descriptions and illustrations. External morphology was observed under a stereoscopic microscope and characters were measured with an ocular micrometer. Measurements are given in millimeters; body length is measured from the apex of the head to the apex of the forewing in repose. The genital segments of the examined specimens were macerated in 10% KOH, washed in water and transferred to glycerin. Illustrations of the specimens were made with a Leica MZ 12.5 stereomicroscope. Photographs were taken with a Leica D-lux 3 digital camera. The digital images were then imported into Adobe Photoshop 8.0 for labeling and plate composition.

Type specimens of the new species here described are deposited in the Institute of Entomology, Guizhou University, Guiyang, China (IEGU).

## Taxonomy

### Key to genera of Mukariini

**Table:** 

1	Apex of head in profile thin and acuminate, ventral part of face flat and lying nearly horizontally (Figs 6, 8)	**2**
–	Apex of head in profile thick and truncate, ventral part of face tumid distally	**5**
2	Aedeagus with single shaft and 1 gonopore	**3**
–	Aedeagus with 2 shafts and 2 gonopores (Figs 21, 23)	**4**
3	Forewing with vein M_3+4_ originating from the central anteapical cell; male pygofer with one process at inside of posterior margin; subgenital plate with a single row of macrosetae; connective V-shaped	***Flatfronta***
–	Forewing with vein M_3+4_ originating from inner anteapical cell; male pygofer with two processes at posterior margin; subgenital plate with several rows of macrosetae; connective Y-shaped	***Tiaobeinia***
4	Hindwing with veins R_4+5_ and M_1+2_ separated basally (Fig. 9); male anal segment with large process ventrally (Figs 16–18)	***Bambusimukaria***
–	Hindwing with veins R_4+5_ and M_1+2_ confluent basally; male anal segment without process ventrally	***Pseudobalbillus***
5	Crown in dorsal view rather short, anterior margin broadly rounded	***Buloria***
–	Crown in dorsal view relatively long, anterior margin acutely rounded	**6**
6	Aedeagus with 2 shafts and 2 gonopores	**7**
–	Aedeagus with single shaft and 1 gonopore	**9**
7	Male pygofer side with process	**8**
–	Male pygofer side without process	***Neobassareus***
8	Body broad and dorsoventrally depressed, black, without longitudinal stripe dorsally; anterior margin of head with several carinae; male pygofer with process at posterior or ventral margin	***Mukaria***
–	Body normal, yellowish white, with dark longitudinal stripe dorsally; anterior margin of head without carina; male pygofer with process at inside of dorsal margin	***Pseudomohunia***
9	Valve and subgenital plates fused	***Agrica***
–	Valve and subgenital plates not fused	**10**
10	Forewing with vein M_3+4_ originating from central anteapical cell	**11**
–	Forewing with vein M_3+4_ originating from inner anteapical cell	**13**
11	Male pygofer with process at posterior margin	***Mohunia***
–	Male pygofer with process at ventral margin	**12**
12	Male pygofer with a single process at inside of ventral margin	***Benglebra***
–	Male pygofer with paired processes at ventral margin	***Scaphotettix***
13	Hindwing with veins R_4+5_ and M_1+2_ confluent basally; connective Y-shaped	***Neomohunia***
–	Hindwing with veins R_4+5_ and M_1+2_ separated basally; connective slender quadrate	***Paramohunia***

**Figures 1–6.**
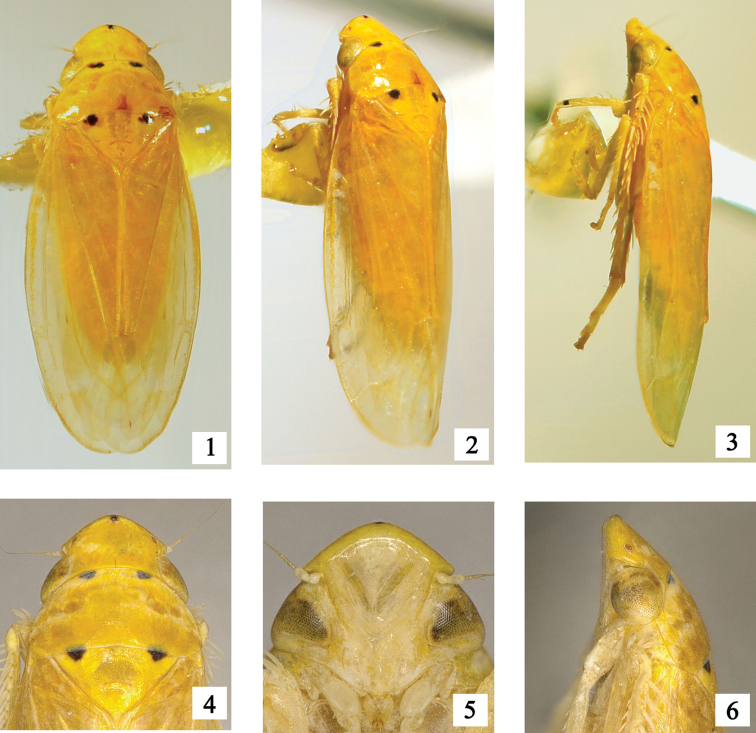
*Bambusimukaria quinquepunctata* sp. n. **1** Male habitus, dorsal view **2** Male habitus, dorsal and lateral view **3** Male habitus, lateral view **4** Head and thorax, dorsal view **5** Face **6** Head and thorax, lateral view.

**Figures 7–14.**
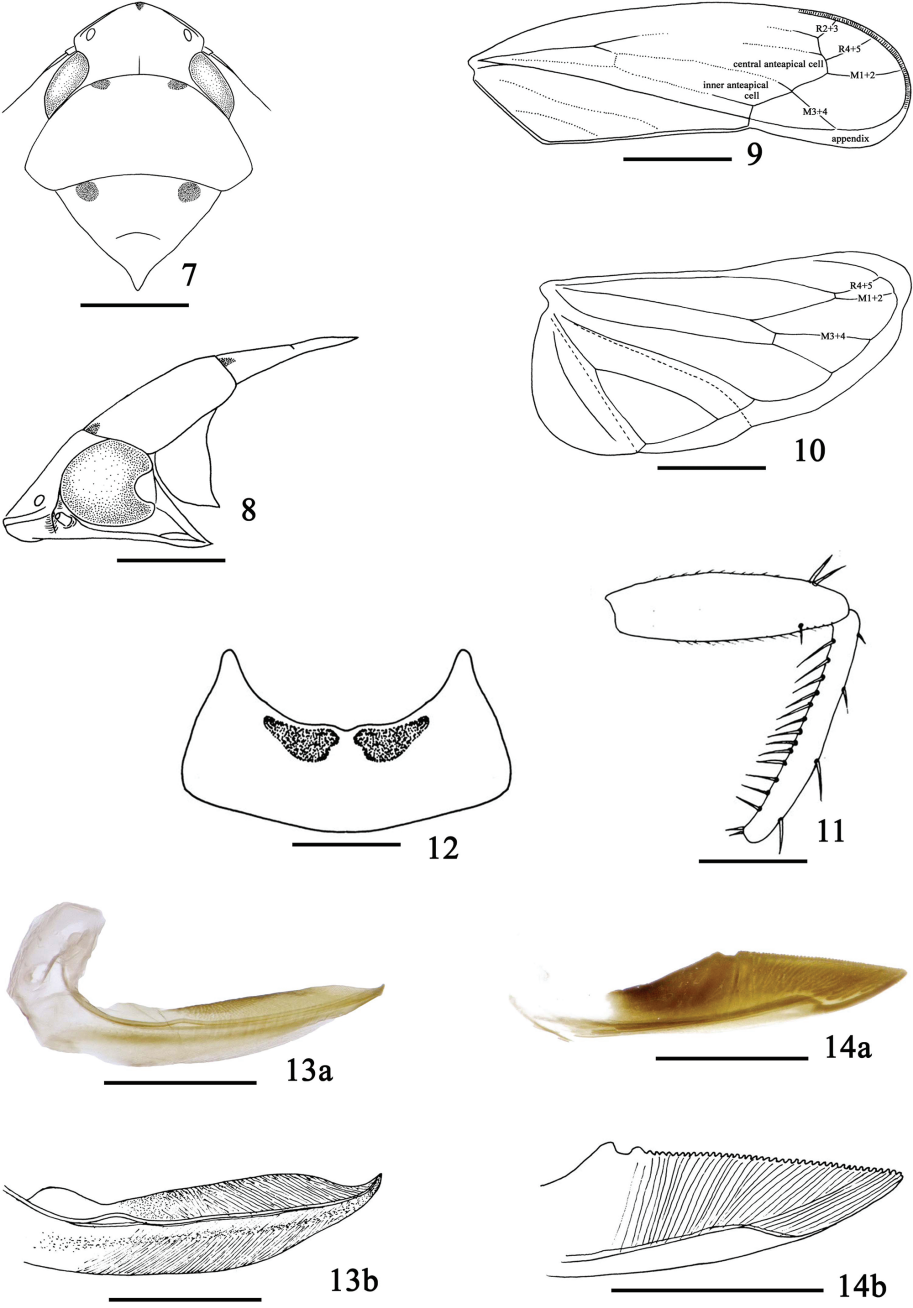
*Bambusimukaria quinquepunctata* sp. n. **7** Head and thorax, dorsal view **8** Head and thorax, lateral view **9** Forewing **10** Hindwing **11** Fore femur and tibia, anterior surface **12** Female sternite VII, ventral view **13a** First valvula and valvifer, lateral view **13b** Apex of first valvula, lateral view **14a** Second valvula, lateral view **14b** Apex of second valvula, lateral view. Scale bars: 1.0 mm (**7–12**); 0.5 mm (**13–14**).

**Figures 15–23.**
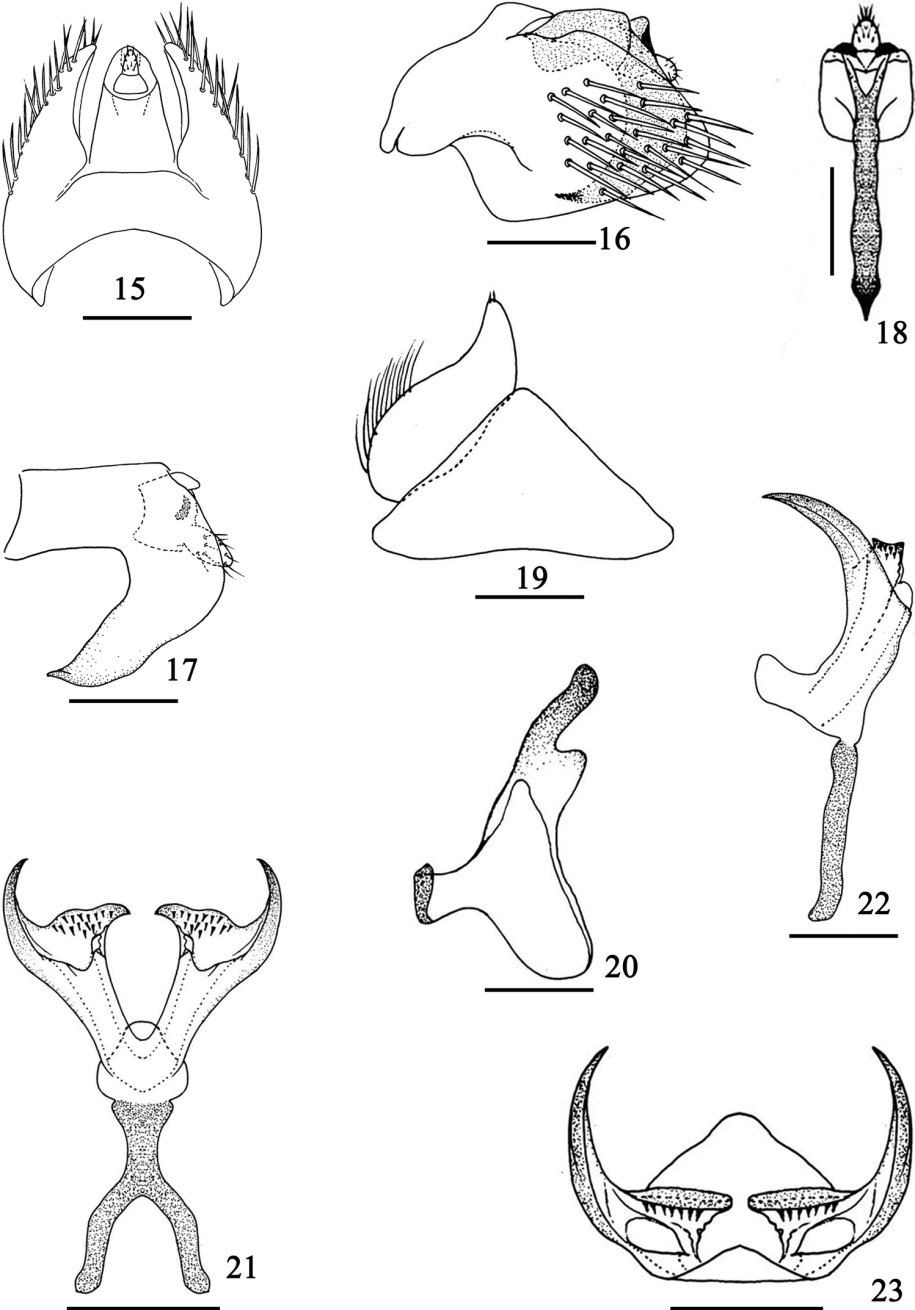
*Bambusimukaria quinquepunctata* sp. n. **15** Pygofer and anal tube, dorsal view **16** Pygofer and anal tube, lateral view **17** Anal tube, lateral view **18** Anal tube, postero-ventral view **19** Valve and right subgenital plate, ventral view **20** Style, dorsal view **21** Aedeagus and connective, ventral view **22** Aedeagus and connective, lateral view **23** Aedeagus, caudal view. Scale bars: 1.0 mm (**15–20**); 0.5 mm (**21–23**).

### Bambusimukaria gen. n.

Taxon classificationAnimaliaHemipteraCicadellidae

http://zoobank.org/F6030468-65D6-48A2-A5B7-3B36477D9DB9

Figs 1–6 , 7–14 , 15–23 , 26 , 27

#### Type species.

*Bambusimukaria quinquepunctata* sp. n., here designated.

#### Diagnosis.

Crown with anterior and submarginal carinae; entire second segment of antenna visible from above. Frontoclypeus transversely impressed across base beneath prominent overhanging anterior edge of head. Forewing with four apical cells, venation obscure except near apex, vein M_3+4_ originating from junction of inner and central anteapical cell. Hind wing with four closed apical cells. Ventral margin of male pygofer without process. Style with short articulating arm and broad outer basal arm. Connective Y-shaped, fused with aedeagus. Aedeagus with paired stout shafts diverging from base, gonopores subapical, large; basal apodeme short.

#### Description.

**Head and thorax.** Crown (Figs 4, 7) shorter than pronotum, subconically anteriorly rounded, more than half as long as breadth between eyes, with anterior and submarginal carinae, posterior end of anterior carina strongly incurved before eyes; disk strongly sloping posteriorly, texture smooth; ocelli on crown, distant from eyes and close to anterior margin; entire second segment of antenna visible from above; eyes long, oblique, extending backward over anterior angles of pronotum; face (Fig. 5) including eyes as long as broad, frontoclypeus transversely impressed across base beneath prominent overhanging anterior edge of head, narrowed towards clypeus; clypellus narrowing apically; lorum broad. Pronotum (Figs 4, 7) elevated centrally, arched, anterior margin convexly rounded between eyes, posterior margin slightly concave, lateral margin short. Scutellum (Figs 4, 7) large, broad, basal margin longer than lateral margin, transverse depression slightly curving. Forewing (Figs 1–3, 9) elongate, considerably longer than abdomen, slightly widened posteriorly, with four apical cells, venation obscure except near apex, vein M_3+4_ originating from junction of inner and central anteapical cell; appendix well developed. Hind wing (Fig. 10) with four closed apical cells. Profemur (Fig. 11) with 2 dorsoapical setae, row AM with 1 stout seta, and row AV with several fine setae. Protibia (Fig. 11) with 4 macrosetae in row AD and with 13 macrosetae approximately equal in length in row AV. Hind femur broadened distally and slightly bowed; apical setal formula 2+2+1. Hind tibia flattened and nearly straight, with PD setae very long, alternating in length and with 1 smaller setae between macrosetae; row AD with 14 macrosetae interspersed by 1 to 2 small stout setae; several supernumeral setae present between AD and AV rows. Metabasitarsomere with 3 platellae and 2 setae on apical transverse row, and one row of 6 stout setae at middle and one row of 4 stout setae at lateral margin.

**Male genitalia.** Male pygofer (Figs 15, 16) rather dorso-ventrally depressed, with macrosetae caudally; ventral margin without process. Valve (Fig. 19) broad, subtriangular. Subgenital plate (Fig. 19) very short, broad basally, with group of moderately long fine setae laterobasally and few short fine setae apically. Style (Fig. 20) with short articulating arm and broad outer basal arm. Connective (Fig. 21) Y-shaped, fused with aedeagus. Aedeagus (Figs 21–23) with paired stout shafts diverging from base, gonopores subapical, large; basal apodeme short, thumb-like in lateral view.

**Female genitalia.** Sternite VII (Fig. 12) with hind margin broadly concave. Pygofer with numerous macrosetae. Ovipositor protruding slightly beyond pygofer apex. First valvula (Fig. 13a, b) weakly curved; dorsal sculpturing pattern strigate, reaching dorsal margin; without distinctly delimited ventroapical sculpturing. Second valvula (Fig. 14a, b) broad, widest near mid-length, thereafter gradually tapered to acute apex; with broad dorsal sclerotized area, thereafter dorsal margin with numerous fine regular teeth after dorsal prominences.

#### Host plant.

**Figures 24–27.**
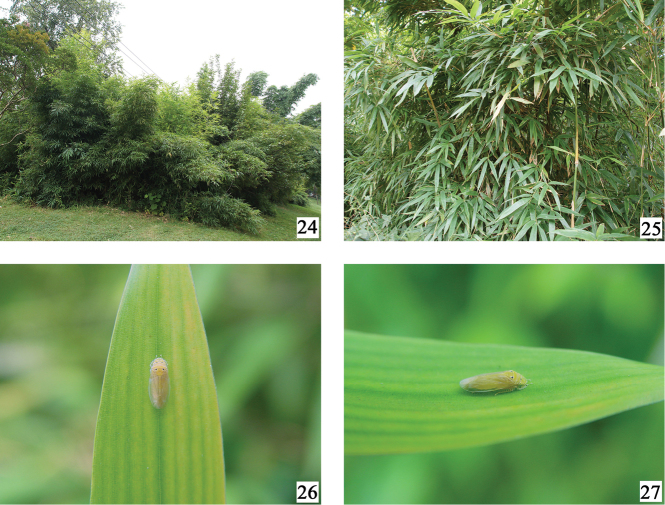
Host plant of *Bambusimukaria quinquepunctata* sp. n. **24** View of the area where the types of *Bambusimukaria quinquepunctata* were captured, in Guiyang Forest Park (Guizhou, China) with Phyllostachys bambusoides f. lacrimadeae Keng & Wen **25** View of the plant **26** *Bambusimukaria quinquepunctata* resting on a leaf of Phyllostachys bambusoides f. lacrimadeae, dorsal view (Guiyang Forest Park, Guizhou) **27** same, lateral view. (11 Aug 2006, photography by X.-S. Chen)

Bamboo (Figs 24–27).

#### Distribution.

Southwest and south China.

#### Etymology.

The genus name, which is feminine, is a combination of “bambus” (bamboo) and “*Mukaria*” (name of the type genus of Mukariini), meaning that members of this genus feeding exclusively on bamboo (Bambusoideae).

#### Remarks.

The new genus can be distinguished from other genera of Mukariini by the very large anal tube process (see also above key to genera of Mukariini). Among other Chinese mukariin genera, the new genus is somewhat similar to *Flatfronta* Chen & Li, 1997 and *Tiaobeinia* Chen & Li, 2008 in the shape of head, and also similar to *Mukaria* Distant, 1908 in the shape of male genitalia. See also Table 1 for further comparisons.

**Table 1.** Morphological comparison of *Bambusimukaria* to similar genera, *Flatfronta*, *Tiaobeinia* and *Mukaria*.

	***Bambusimukaria***	***Flatfronta***	***Tiaobeinia***	***Mukaria***
Body form	Depressed	Depressed	Depressed	Weakly depressed
No. of carinae on crown	Two	One	One	Two or three
Anterior margin of crown in dorsal view	Strongly incurved before eyes	Smoothly curved	Smoothly curved	Smoothly curved
Disk of crown	Strongly elevated posteriorly	Weakly elevated posteriorly	Weakly elevated posteriorly	Strongly elevated posteriorly
Frontoclypeus form	Mainly flat	Mainly flat	Mainly flat	Tumid anteriorly and depressed posteriorly
Forewing vein M_3+4_ originating from	Inner anteapical cell	Central anteapical cell	Inner anteapical cell	Inner anteapical cell
Hindwing veins R_4+5_ and M_1+2_	Separated basally	Confluent basally	Separated basally	Separated basally
Hind femur macrosetae	2+2+1	2+2+1	2+2+1+1	2+2+1
Pygofer process	Absent	Present	Present	Present or absent
Subgenital plate macrosetae	Absent	One row	Several rows	Absent
Connective form	Y-shaped	V-shaped	Y-shaped	U-shaped
Ventral process of anal segment	Present	Absent	Absent	Absent
Number of gonopores	Two	One	One	Two

### Bambusimukaria quinquepunctata sp. n.

Taxon classificationAnimaliaHemipteraCicadellidae

http://zoobank.org/A5330454-C791-40F9-8BAC-9FCEFD88EFEF

Figs 1–6 , 7–14 , 15–23 , 26 , 27

Bambusimukaria quinquepunctatus , in press, Chen et al. (2012).

#### Type material.

Holotype: ♂, **China**: Forest Park (26°35'N, 106°42') (1100 m), Guiyang, Guizhou, on bamboo (*Phyllostachys bambusoides*), 11 Aug. 2006, X.-S. Chen and L. Yang; paratypes: 4♂♂, 7♀♀, data same as holotype; 1♀, Dongtang (25°24'N, 107°52'), Maolan, Libo, Guizhou, on bamboo, 24 May 1998, X.-S. Chen; 10♀♀, Dayi (25°21'N, 106°06'), Wangmo, Guizhou, on bamboo (*Phyllostachys bambusoides*), 28 July 1998, X.-S. Chen; 25♂♂, 6♀♀, Forest Park, Guiyang, Guizhou, on bamboo, 11 July 2006, Q.-Z. Song; 1♀, Weiyuan (26°01'N, 106°31'), Changshun, Guizhou, on bamboo, 11 July 2007, X.-S. Chen; 6♀♀, Daxianfeng (26°55'N, 116°59'), Datian, Sanming, Fujian, on bamboo, 14 May 2011, Z.-M. Chang and J.-K. Long; 5♀♀, Tianyanbao (26°39'N, 118°53'), Yongan, Fujian, on bamboo, 17 May 2011, Z.-M. Chang and W.-C. Yang. All types are deposited in IEGU except two males and two females deposited in BMNH where indicated.

#### Diagnosis.

General color yellowish white to yellowish orange. Head and thorax with five black markings. Female sternite VII with two blackish brown markings. Anal (Xth) segment with a very large process at apical-ventral margin. Aedeagus with shafts diverging from base, each shaft narrower at base, broad to near apex, outer margin extended apically into a stout acute process inner margin with a stout subapical tooth-like process directed medially, dentate on dorsal suface, gonopores subapical on ventral surface.

#### Description.

**Measurements.** Body length including forewing: male 5.30–5.40 mm (n = 30), female 5.50–5.60 mm (n = 36).

**Coloration.** General color yellowish white to yellowish orange (Figs 1–6, 26, 27). Eyes yellowish brown. Head and thorax (Figs 4, 7) with five black markings, one at apex of crown, two on anterior margin of pronotum and two on anterior margin of mesonotum. Fore tibia with one dark brown mark subapically. Female sternite VII with two blackish brown markings (Fig. 12).

**Head and thorax.** Crown (Figs 4, 7) with median length shorter than width between eyes (0.62:1). Face including eyes (Fig. 5) slightly shorter in middle line than broad at widest part (0.81:1). Pronotum (Figs 4, 7) wider than head including eyes (1.17:1), longer than vertex in middle line (1.48:1). Scutellum (Figs 4, 7) as long as pronotum in middle. Forewing (Fig. 9) 3.4 times longer in middle line than widest part. Hindwing (Fig. 10) 2.13 times longer in middle than widest part.

**Male genitalia.** Anal (Xth) segment (Figs 15–18) with a very large process at apical-ventral margin, directed cephalad, tapering distally to acute apex. Pygofer (Figs 15, 16) broad and rounded in lateral view, with many macrosetae. Valve (Fig. 19) with basal width 2 times longer than median length, posterior margin rounded. Subgenital plate (Fig. 19) very short, broad at base, tapering to acutely rounded apex. Style apophysis (Fig. 20) thumb-like, slightly sinuate, apex rounded. Connective stem (Figs 21, 22) slightly shorter than arms, fused with base of aedeagus. Aedeagus (Figs 21–23) in ventral view with shafts diverging from base, each shaft narrower at base, broad to near apex, outer margin extended apically into a stout acute process inner margin with a stout subapical tooth-like process directed medially, dentate on dorsal suface, gonopores subapical on ventral surface.

**Female genitalia.** Sternite VII (Fig. 12) with anterior margin angularly produced laterally, posterior margin strongly and broadly concaved. First and second valvulae (Fig. 13a, b) as in generic description; second valvulae (Fig. 14a, b) bearing approximately 36 fine teeth on apical half behind dorsal prominence and basal curvature.

#### Host plant.

Bamboo (Phyllostachys bambusoides f. lacrimadeae Keng *et* Wen) (Figs 24–27).

#### Distribution.

Southwest and south China (Guizhou, Fujian).

#### Etymology.

The name is a combination of the Latin words “quinque” (five) and “punctata” (spots), which refers to the dorsum of head and thorax with five small dark spots.

#### Remarks.

The new species can be distinguished from other species of Mukariini by the very large anal tube process.

## Supplementary Material

XML Treatment for Bambusimukaria

XML Treatment for Bambusimukaria quinquepunctata
